# Evaluation of Stability and Biological Activity of Solid Nanodispersion of Lambda-Cyhalothrin

**DOI:** 10.1371/journal.pone.0135953

**Published:** 2015-08-17

**Authors:** Bo Cui, Lei Feng, Zhenzhong Pan, Manli Yu, Zhanghua Zeng, Changjiao Sun, Xiang Zhao, Yan Wang, Haixin Cui

**Affiliations:** 1 Institute of Environment and Sustainable Development in Agriculture, Chinese Academy of Agricultural Sciences, Beijing, China; 2 College of Natural Resources and Environment, College of Plant Science, Jilin University, Changchun, China; Ghent University, BELGIUM

## Abstract

Pesticides are essential agrochemicals used to protect plants from diseases, pests and weeds. However, the formulation defects of conventional pesticides cause food toxicity and ecological environmental problems. In this study, a novel, efficient and environmentally friendly formulation of lambda-cyhalothrin, a solid nanodispersion, was successfully developed based on melt-emulsification and high-speed shearing methods. The solid nanodispersion presented excellent advantages over conventional pesticide formulations in such formulation functions as dispersibility, stability and bioavailability. The formulation is free of organic solvents, and the use of surfactant is reduced. Therefore, the application of the solid nanodispersion in crop production will improve efficacy and reduce the occurrence of both pesticide residues in food and environmental pollution from pesticides.

## Introduction

Pesticides are essential agrochemicals widely used for plant protection and to decrease production loss. However, pesticides also cause numerous ecological and environmental problems, such as the presence of residues in food, soil and waterbodies, and a reduction in biodiversity due to their low efficacy and overuse [1–4]. Most pesticide compounds are poorly soluble in aqueous media, which inhibits the construction of pesticide formulations with high efficacy and safety [5].

Lambda-cyhalothrin is a halogenated pyrethroid comprising a 1:1 mixture of two stereoisomers, (S)-α-cyano-3-phenoxybenzyl-(Z)-(1R,3R)-3-(2-chloro-3,3,3-trifluoroprop-1-enyl)-2,2-dimethylcyclopropanecarboxylate and (R)-α-cyano-3-phenoxybenzyl-(Z)-(1S,3S)-3-(2-chloro-3,3,3-trifluoroprop-1-enyl)-2,2-dimethylcyclopropanecarboxylate. It is a poorly water-soluble (5 ug/L at 21°C) pesticide compound used as a broad-spectrum insecticide with high toxicity to many kinds of pests [6,7]. The conventional formulations of lambda-cyhalothrin mainly include wettable powder (WP), emulsifiable concentrate (EC) and emulsion in water (EW). These formulations have some disadvantages, including dust drift, pollution resulting from the use of organic solvent and poor dispersion, further decreasing their pest control efficacy [8–10].

Assuming a spherical particle, the dependence of solubility on particle size can be described by the Ostwald–Freundlich equation [11,12]:

$$\text{ln}(\text{S}/{\text{S}}_{0})=(2\text{M}\gamma )/(\rho \text{rRT})$$

where S and S_0_ are the solubilities of a particle of radius r and of infinite size, respectively. γ, M and ρ are interfacial tension at the particle surface, the compound molecular weight, and the density of the particle, respectively. R is the gas constant, and T is the temperature. Therefore, shrinking particles to the nano scale will improve the solubility and dispersibility of poorly soluble pesticides in water, and further improve their bioavailability.

Nano-pesticide formulations with nanoparticles as delivery systems possess some highly desirable traits, such as increased saturation solubility, wettability, penetration, adhesion to the surface of crop leaves and targeted insects, and biological activity due to the effect of their small size and high surface area [13,14]. Microemulsions are currently regarded as a new type of nano-formulation that overcomes the above disadvantages of conventional pesticide formulations. However, microemulsions usually need a large amount of surfactant to emulsify pesticide compounds in water, which may lead to some food safety risks and environmental issues [15,16]. Therefore, it is important and urgent to develop a new type of nano-pesticide formulation with higher efficacy and environmental friendliness.

Recently, nanosuspensions, consisting of drug nanoparticles less than 1 m in diameter suspended in aqueous medium using surfactants as stabilizers, have attracted a large amount of attention as an effective formulation in pharmaceutics [17–19]. Most nanosizing approaches to obtain nanosuspensions involve bottom-up methods (such as microprecipitation and supercritical fluid methods) or top-down methods (such as wet-milling and high pressure homogenization) [19–23]. However, these methods suffer from the need for special high-cost equipment, in-process heat generation and the heavy use of surfactants, which limit the application of nanosuspensions in the field of pesticides. By contrast, a solid nanodispersion is a solidified nano-formulation with hydrophobic pesticide nanoparticles dispersed in a solid hydrophilic matrix. Solid nanodispersions can maintain the desirable properties of nanosuspensions while demonstrating improved stability and safety during storage and transportation. Nevertheless, the relevant research in the field of pesticide science is still in nascent stage.

In this present investigation, a solid nanodispersion of lambda-cyhalothrin was prepared by a combination of melt-emulsification and high-speed shearing, and characterized with respect to crystallinity, suspensibility, wettability, stability and biological activity. This formulation avoided the use of any organic solvent and substantially reduced the surfactant content relative to conventional formulations, improving the safety and environmental friendliness of the pesticide.

## Materials and Methods

### 2.1. Materials

Lambda-cyhalothrin (96%) was purchased from Yangnong Chemical Co., Ltd. (Jiangsu, China). 1-Dodecanesulfonic acid sodium salt (SDS), polyvinylpyrrolidone K30 (PVP K30), hexadecyltrimethylammonium bromide (CTAB), sodium ligninsulfonate (SL), polyoxyethylene sorbitan monooleate (Tween 80) and sucrose were purchased from J&K Scientific Ltd. (Beijing, China). Sodium dodecylbenzenesulfonate (SDBS) and hydroxypropyl methylcellulose (HPMC) were obtained from Sigma-Aldrich Shanghai Trading Co., Ltd. (Shanghai, China). Maleic rosin-polyoxypropylene-polyoxyethylene ether sulfonate (MRES) and polycarboxylate were provided by Sinvochem S&D Co., Ltd. (Jiangsu, China). The EC and EW of lambda-cyhalothrin were purchased from Hengtian Chemical Co., Ltd. (Weinan, China). All the chemicals were used as received. The water used in all analytical experiments was Milli-Q water (18.2 MΩ.cm, TOC ≤ 4 ppb) prepared by a Milli-Q Advantage A10 system (Millipore, Milford, MA, USA).

### 2.2. Preparation of the Lambda-Cyhalothrin Solid Nanodispersion

The lambda-cyhalothrin solid nanodispersion was prepared by a combination of melt-emulsification and high-speed shearing [24]. First, the aqueous dispersions of lambda-cyhalothrin and surfactants were mixed, melted and emulsified by a shearing machine (C25, ATS Engineering Ltd., Vancouver, Canada) to produce a nanosuspension. Next, sucrose as a water-soluble carrier was added slowly, followed by shearing at 10000 rpm for 10 min. Finally, the water was removed using a freeze drier (FD-81, EYELA, Tokyo, Japan) to obtain a solid nanodispersion of lambda-cyhalothrin.

### 2.3. Determination of the Particle Size and Zeta Potential

The particle size, polydispersity index (PDI) and zeta potential of the samples were determined by dynamic light scattering (DLS) using a Zetasizer Nano ZS 90 (Malvern, Worcestershire, UK). Particle size was expressed by the mean size and 90% (D90) diameter percentile. PDI values larger than 0.5 indicate a very broad size distribution. Zeta potentials above an absolute value of 30 mV indicate that the suspension has good stability. Each sample was measured in triplicate.

### 2.4. Morphological and Structural Characterization of the Nanoparticles

The morphology of the nanoparticles was monitored by a scanning electron microscope (SEM) (JSM-7401F, JEOL, Tokyo, Japan) at 3 kV. A single drop of the aqueous solution with dispersed nanoparticles was deposited onto a freshly cleaned silicon slide. The samples were air-dried and then platinum coated (thickness ≤ 2 nm) with a sputter coater (Beijing Elaborate Technology Development Ltd., Beijing, China) using an electric current of 2 mA for 3 min. The scale bar was calibrated accurately.

X-ray diffraction (XRD) was used to assess the degree of crystallinity of the samples. The patterns were generated by a diffractometer (D8 ADVANCE, Bruker AXS Inc., Karlsruhe, Germany) using CuKα radiation with the following measurement conditions: tube voltage of 40 kV, tube current of 40 mA, step scan mode with a step size of 2θ = 0.02°, and counting time of 0.1 s per step.

### 2.5. Determination of Lambda-Cyhalothrin Content

The pesticide content of the formulation was determined by high performance liquid chromatography (HPLC) (Waters 2695, Waters Co., Milford, MA, USA) using a C18 column (5 um, 4.6 mm*150 mm, Waters Co., Milford, MA, USA) at room temperature. The mobile phase was composed of methanol and water (80:20). The flow rate was 1.0 ml/min, and a UV detector wavelength of 278 nm was used.

### 2.6. Suspensibility Test

The lambda-cyhalothrin solid nanodispersion was added slowly to a beaker containing 50 ml standard hard water (30 ±1°C). After swirling by hand in a circular motion at a rate of approximately 120 times per minute for 2 min, the suspension was placed in a 30 ±1°C constant temperature bath for 4 min. The solution was then transferred to a 250-ml measuring cylinder. Subsequently, 200 ml standard hard water was used to rinse the beaker, and the cylinder was filled to scale. The measuring cylinder was then stoppered and inverted 30 times by hand and placed in the 30°C water bath in an upright position free from vibration. After standing for 30 min, the top 225 ml of the solution was removed. The drug contents of the original suspension and the remaining 25 ml of solution were measured by HPLC.

### 2.7. Wettability Test

The solid nanodispersion was poured into standard hard water held at 25 ±1°C. A stopwatch was used to measure the time elapsed from the instant of the pouring until the point at which the powder was entirely wetted by the water. The test was repeated three times, and the average value was used.

The wettability of the formulation on leaf surfaces was investigated based on contact angle measurements. The measurements were implemented with an OCA20 machine (Data Physics, Filderstadt, Germany) at ambient temperature. Droplets of aqueous solution with dispersed lambda-cyhalothrin solid nanodispersion (2 μL) were dropped carefully onto the leaves. The average value of five measurements was adopted.

### 2.8. Bioassays

Bioassays were conducted using the leaf-dip method. Rape (*Brassica campestris* L.) leaves were immersed in aqueous solutions of pesticides with different concentrations containing 0.05% Triton X-100 for 10 s. The leaves were air-dried and then placed in a culture dish with a filter paper. Ten second-instar diamondback moth (*Plutella xylostella* L.) larvae were introduced into each dish, and three replications were conducted for comparison with the control test (leaves treated only with 0.05% Triton X-100 solution). Mortality was assessed at 48 h after treatment. Concentration–mortality data were analysed using DPS 8.1 (Refine Information Tech. Co., Ltd., Hangzhou, China). The median lethal concentrations (LC 50) and their 95% confidence limits were also calculated.

## Results and Discussion

The lambda-cyhalothrin solid nanodispersion was prepared by a combination of melt-emulsification and high-speed shearing. Because the particle size and distribution of the nanosuspension before solidification have a significant impact on the properties of the final solid nanodispersion, the formulation composition and preparation parameters of the nanosuspension were investigated in detail using the particle size and polydispersity index (PDI) as evaluation indices.

### 3.1. Surfactant Screening and Composition Optimization

The effects of three kinds of surfactants on the particle size and dispersibility of the nanosuspension were investigated using mean particle size, D90 and PDI as indices. The experimental results are shown in Table 1. Generally, the effects of the anionic surfactants (SDS, SDBS, MRES, SL and polycarboxylate) were superior to those of cationic (CTAB) and nonionic (Tween 80, PVP K30 and HPMC) surfactants. Among the nine surfactants, SDS, SDBS and MRES reduced the mean particle size of the pesticide to less than 100 nm. D90 is a very sensitive parameter for quantifying large particles, and PDI values lower than 0.3 indicate a narrow size distribution. The large D90 and PDI of the SDBS-modified pesticide particles suggested that the nanosuspension was inhomogeneous and the solution contained large aggregates, which would decrease the system’s stability by Ostwald ripening. Both negatively charged MRES and SDS could stabilize the pesticide nanoparticles and improve the dispersibility of the nanosuspension by electrostatic repulsion. Furthermore, it has been reported that steric barriers can effectively inhibit particle growth in suspensions [25]. SDS was capable of reducing particle size significantly, whereas polymeric MRES has long hydrophobic chain and could induce steric effect to prevent aggregation. Therefore, given the complementarity of SDS and MRES in particle size reduction and dispersibility improvement, the combination of SDS and MRES was chosen to prepare lambda-cyhalothrin nanodispersions, and the appropriate proportion of these two components was further investigated.

**Table 1:** Effect of surfactants on the particle properties of lambda-cyhalothrin nanosuspensions.^a^

Surfactant^b^	Mean size (nm) ± S.D.	D90^c^ (nm) ± S.D.	PDI^d^ ± S.D.^e^
SDS	19.7 ± 0.1	93.9 ± 3.5	0.38 ± 0.01
SDBS	37.7 ± 1.1	325.3 ± 93.6	0.49 ± 0.01
MRES	81.9 ± 0.4	157.0 ± 6.0	0.19 ± 0.02
SL	431.0 ± 20.1	733.3 ± 335.8	0.23 ± 0.14
Polycarboxylate	481.6 ± 16.8	794.7 ± 381.2	0.39 ± 0.35
CTAB	179.9 ± 2.3	579.3 ± 186.6	0.27 ± 0.01
Tween 80	117.3 ± 1.2	286.0 ± 33.8	0.24 ± 0.01
PVP K30	1452.8 ± 475.4	385.7 ± 242.5	0.95 ± 0.09
HPMC	1204.7 ± 23.2	4443.3 ± 938.3	0.58 ± 0.19

^a^The nanosuspensions containing 1% (w/w) lambda-cyhalothrin and 0.5% (w/w) surfactant were prepared at a shearing speed of 10000 rpm and 80°C.

^b^SDS: 1-dodecanesulfonic acid sodium salt; SDBS: sodium dodecylbenzenesulfonate; MRES: maleic rosin-polyoxypropylene-polyoxyethylene ether sulfonate; SL: sodium ligninsulfonate; CTAB: hexadecyltrimethylammonium bromide; Tween 80: polyoxyethylene sorbitan monooleate; PVP K30: polyvinylpyrrolidone K30; HPMC: hydroxypropyl methylcellulose.

^c^D90: particle size expressed by the 90% diameter percentile.

^d^PDI: polydispersity index.

^e^S.D.: standard deviation of three measurements.

As shown in Table 2, the particle size of the lambda-cyhalothrin nanosuspension decreased as the ratio of MRES to SDS in the mixture increased. The mean particle size and PDI at the optimal ratio of 3:1 (w/w) were 16.2 nm and 0.29, respectively, which illustrated that the combination of MRES and SDS was more effective than any single surfactant in the preparation of a lambda-cyhalothrin nanosuspension with a narrow size distribution. The hydrophobic parts of the two anionic surfactants interacted with the lambda-cyhalothrin molecules and created a negative charge on the surface of the pesticide nanoparticles to prevent aggregation, while the polymeric MRES further enhanced the stability of the dispersion by steric hindrance.

**Table 2:** Effect of the ratio of MRES to SDS on the particle properties of lambda-cyhalothrin nanosuspensions.^a^

Ratio of MRES^b^ to SDS^c^	Mean size (nm) ± S.D.	D90^d^ (nm) ± S.D.	PDI^e^ ± S.D.^f^
1:3	39.3 ± 0.4	230.7 ± 32.3	0.44 ± 0.02
1:1	29.7 ± 0.2	146.7 ± 17.0	0.41 ± 0.01
3:1	16.2 ± 0.1	57.8 ± 5.9	0.29 ± 0.01

^a^The nanosuspensions containing 5% (w/w) lambda-cyhalothrin and 0.25% (w/w) surfactants were prepared at a shearing speed of 10000 rpm and 80°C.

^b^MRES: maleic rosin-polyoxypropylene-polyoxyethylene ether sulfonate.

^c^SDS: 1-dodecanesulfonic acid sodium salt.

^d^D90: particle size expressed by the 90% diameter percentile.

^e^PDI: polydispersity index.

^f^S.D.: standard deviation of three measurements.

**Fig 1:**
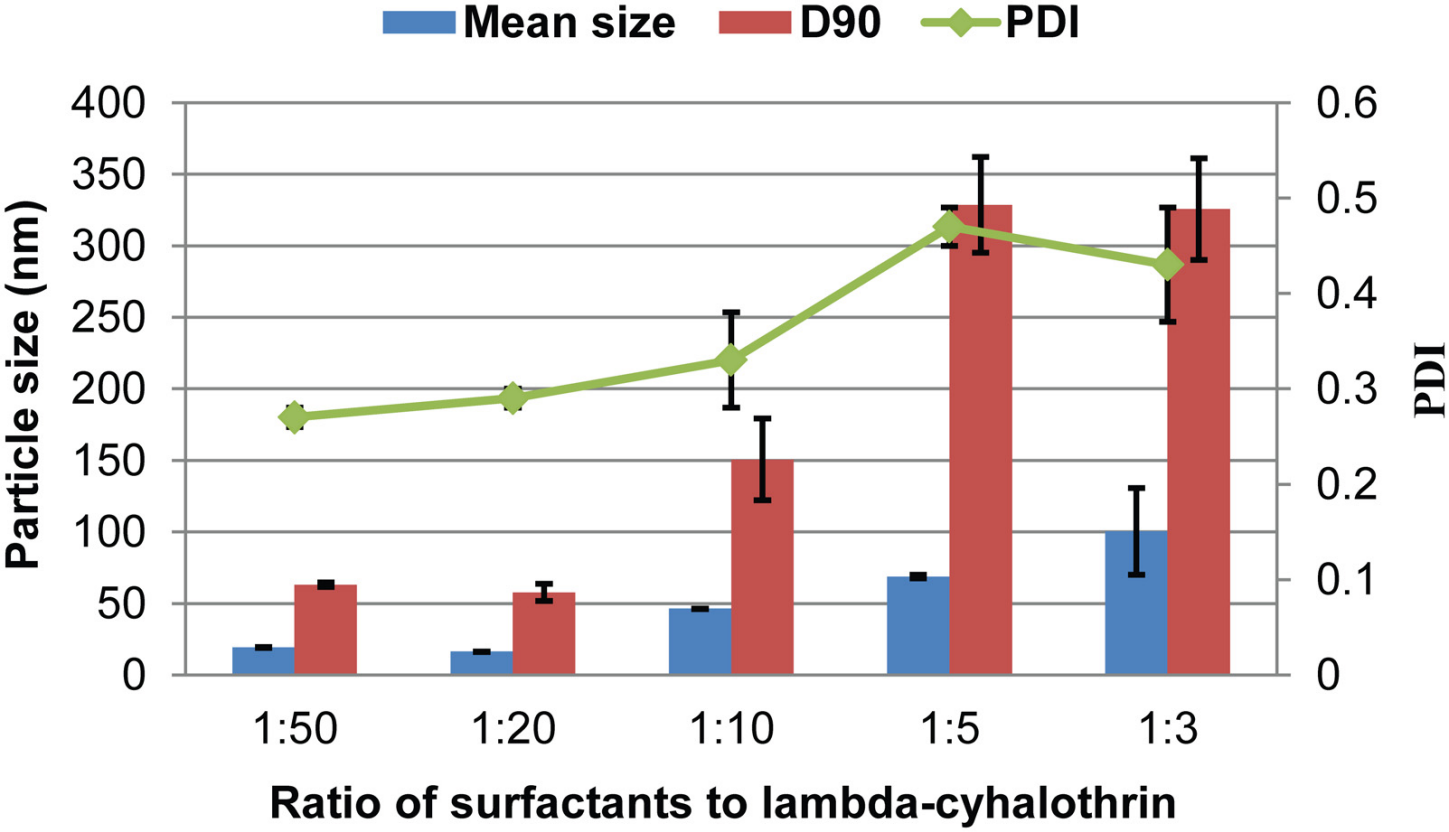
Particle properties of nanosuspensions containing 5% (w/w) lambda-cyhalothrin with different surfactant-to-pesticide ratios. D90: particle size expressed by the 90% diameter percentile; PDI: polydispersity index.

Fig 1 shows the effect of the surfactant-to-pesticide ratio on the particle size and distribution of the nanosuspensions. As the ratio increased from 1:50 to 1:20, the mean size and D90 of the pesticide nanoparticles decreased. However, the mean particle size continuously increased as the surfactant ratio increased from 1:20 to 1:3. This result demonstrates that pesticide nanoparticles exhibited the smallest size and narrow distribution at the 1:20 ratio of surfactant to lambda-cyhalothrin. As the surfactant proportion increases, the neighbouring nanoparticles may aggregate owing to the entanglement of long alkyl chains of polymeric MRES by intermolecular interactions [26,27]. The optimized surfactant content in the nanodispersion was much lower than that in most microemulsion and solid microemulsion formulations [28–30].

### 3.2. Preparation Parameter Optimization

**Fig 2:**
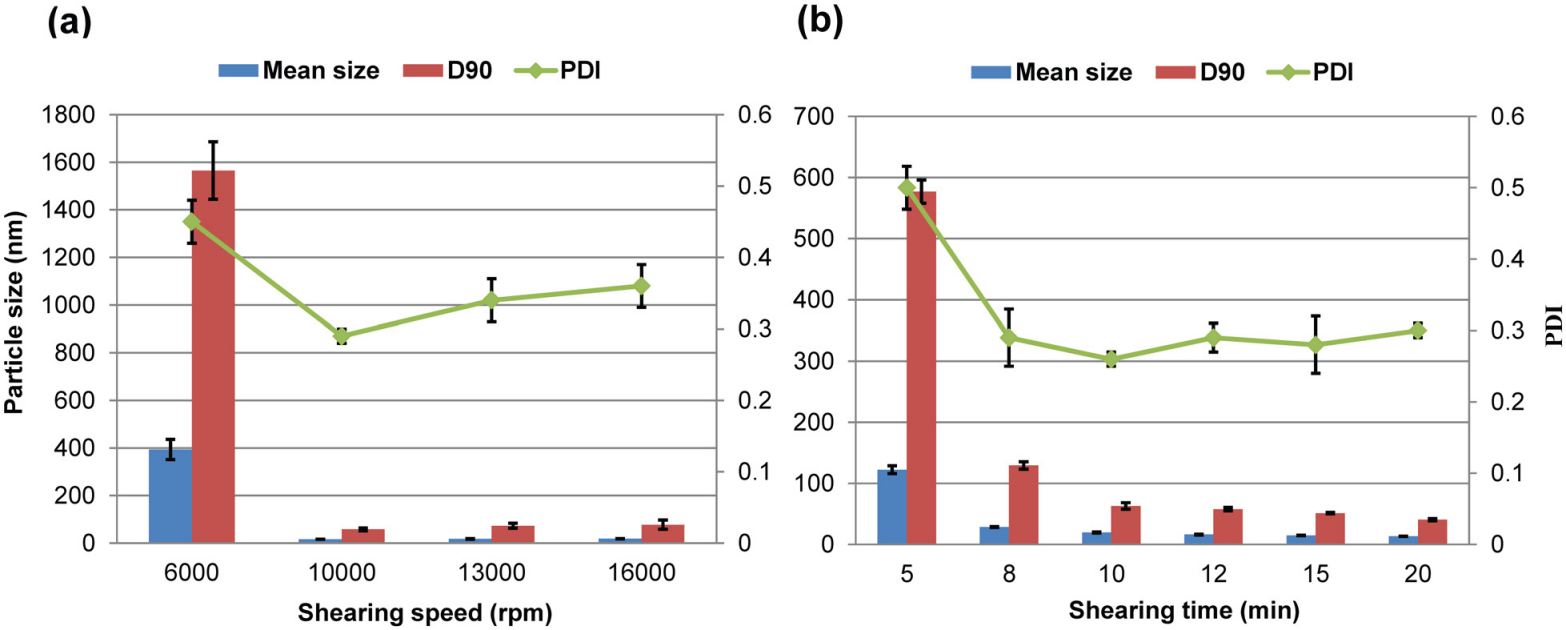
Effect of the shearing process on the particle properties of 5% (w/w) lambda-cyhalothrin nanosuspensions. D90: particle size expressed by the 90% diameter percentile; PDI: polydispersity index; rpm: revolutions per minute.

In this experiment, the process parameters of the melt-emulsification and high-speed shearing methods were investigated, including the shearing speed, shearing time and melting temperature. Fig 2 reveals the effects of shearing speed and time on the particle size and dispersibility of the nanosuspensions. Obviously, the mean size, D90 and PDI of the nanoparticles produced at 6000 rpm were biggest (Fig 2a). This result shows that the production of a nanosuspension with a narrow size distribution requires sufficient energy input, but the conditions remain milder than high pressure homogenization. As shown in Fig 2a, for speeds at or above 10000 rpm, there was no significant difference in mean particle size (approximately 17 nm); nevertheless, the PDI of the 10000-rpm shearing system was smaller than that of the solutions produced at 13000 and 16000 rpm. Therefore, the shearing speed of 10000 rpm was considered to be an appropriate condition for preparing lambda-cyhalothrin nanoparticles. Fig 2b shows that the mean size and D90 of the nanoparticles decreased as the shearing time prolonged at a constant shearing speed of 10000 rpm. After the shearing time exceeding 10 min, the particle size changed little. Therefore, considering the nanosizing effect and energy efficiency, the shearing time of 10 min was chosen.

**Fig 3:**
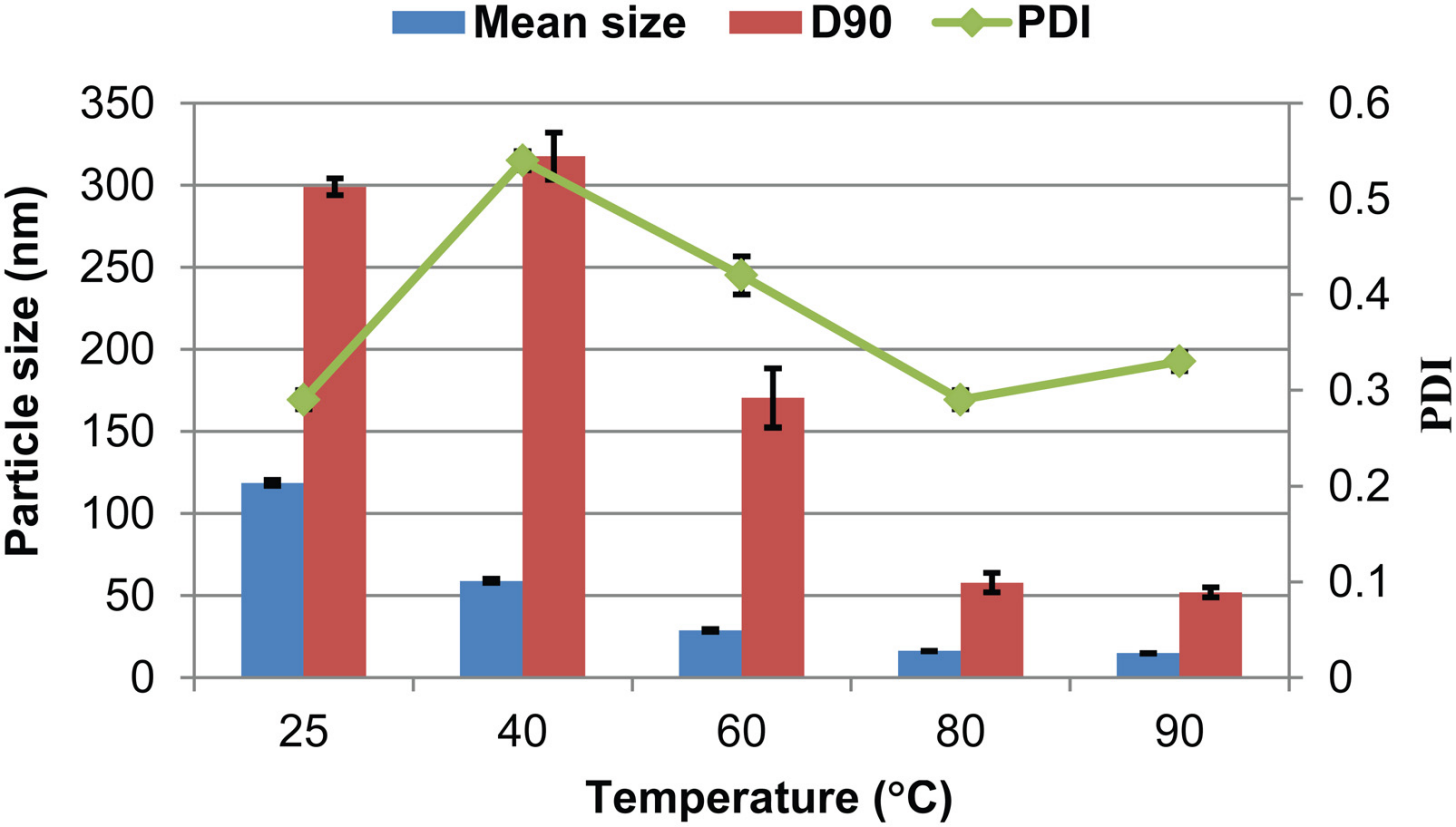
Particle properties of 5% (w/w) lambda-cyhalothrin nanosuspensions prepared at different temperatures. D90: particle size expressed by the 90% diameter percentile; PDI: polydispersity index.

Fig 3 shows the influence of temperature on the produced nanoparticles. The results reveal that the particle size decreased as the temperature increased. The mean size of the pesticide particles declined significantly to 28.7 nm above the melting point of lambda-cyhalothrin (49.2°C). According to previous reports, a melting process, such as melt agglomeration, can improve the dispersion uniformity of a drug in solid dispersion and enhance its dissolution rate [31,32]. In this investigation, the particle size reduction resulting from raising the temperature above 49.2°C was due to the melting of the pesticide, which then gathered into oily droplets that were subsequently diminished into smaller droplets by high shearing forces. These smaller droplets were easily encapsulated and dispersed by the surfactants. In addition, the particle size of the nanosuspension changed little above 80°C. This result further confirms that temperature affected the particle size of the nanosuspensions by changing the pesticide’s physical properties. Ultimately, 80°C was chosen for the preparation of lambda-cyhalothrin nanoparticles.

**Fig 4:**
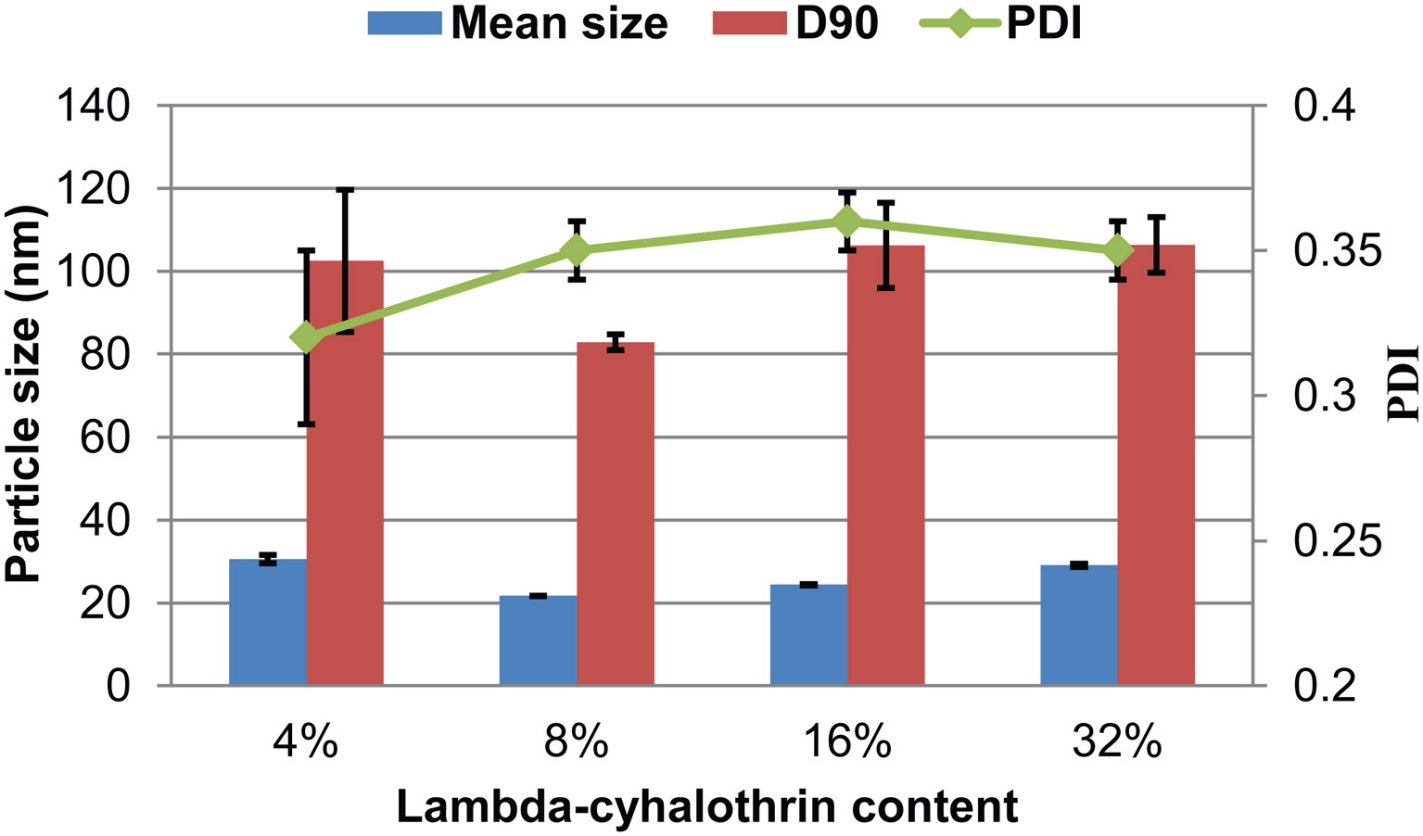
Particle properties of four lambda-cyhalothrin solid nanodispersions with different pesticide contents. D90: particle size expressed by the 90% diameter percentile; PDI: polydispersity index.

To improve the physical stability of the formulation and prolong its shelf life, the nanosuspensions were transformed into solid nanodispersions by lyophilization. Lyophilization has been widely used to produce drug powders from solutions in the pharmaceutical field, and it has proven to be more conducive to preventing particle aggregation than spray-drying method [14,26]. Sucrose is a commonly used carrier and can also be used as an antifreeze agent to protect suspensions from freezing and desiccation impairment [11]. In this study, sucrose as a water-soluble carrier exhibited excellent performance in terms of maintaining the particle size and distribution of the product. As shown in Fig 4, all four solid nanodispersions with different pesticide contents studied were characterized by a mean size and D90 of less than 32 nm and 110 nm, respectively. This clarified that combining the melt-emulsification plus high-speed shearing with freeze-drying is an efficient way to construct a uniform solid nanodispersion, and the pesticide loading in the solid formulation could easily be adjusted by the amount of carrier added.

### 3.3. Characterization of the Solid Nanodispersion

Based on the above research, the optimized preparation process for a lambda-cyhalothrin solid nanodispersion was as follows. First, a lambda-cyhalothrin aqueous dispersion was added to an aqueous solution containing MRES and SDS surfactants (3:1, w/w) at a 1:20 surfactant-to-pesticide ratio. The mixed solution was heated to 80°C and sheared at a rate of 10000 rpm for 10 min to produce a nanosuspension. Next, keeping the bath at 80°C and stirring at 1100 rpm, sucrose was added slowly followed by shearing at 10000 rpm for 10 min. Finally, the dispersion was solidified by lyophilization to obtain a solid nanodispersion.

#### 3.3.1. Size and Morphology

**Fig 5:**
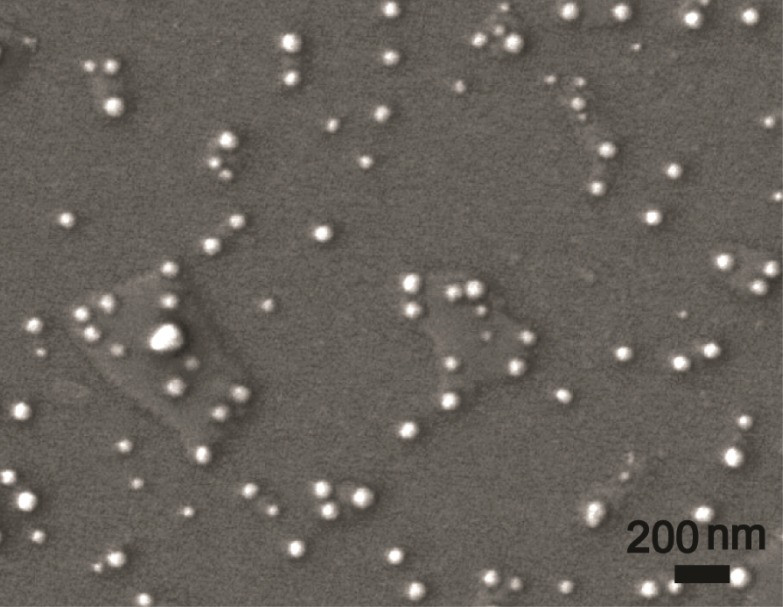
SEM photograph of lambda-cyhalothrin nanoparticles.

As an example, the solid nanodispersion containing 8% (w/w) lambda-cyhalothrin with 0.4% (w/w) surfactant was subjected to a detailed characterization. The mean size and D90 of the re-dispersed aqueous solution of the formulation measured by DLS were 21.7±0.1 nm and 82.8±0.9 nm, respectively. According to the SEM image, the nanoparticles were spherical, and the particle size was in the range of 25 nm to 85 nm (Fig 5), agreeing well with the DLS results. The particles of the solid nanodispersion are significantly smaller than the particles obtained by the solidification of microemulsions and nanosuspensions prepared by high pressure homogenization [20,26,33–35]. This result proves that melt-emulsification combined with high-speed shearing is an effective method for constructing pesticide nano-formulations. A suspension with an absolute zeta potential value higher than 30 mV is always considered stable [36]. However, good stability of a suspension with lower zeta potential can also be obtained when the surfactant provides steric stabilization in addition to electrostatic repulsion [20,37,38]. In this study, the zeta potential and pH of the re-dispersed nanosuspension were– 47 mV and 7.0, respectively. The high zeta potential value indicates the strong stabilizing effect of electrostatic repulsion, and under neutral conditions, the pesticide did not decompose easily.

#### 3.3.2. Crystallinity

**Fig 6:**
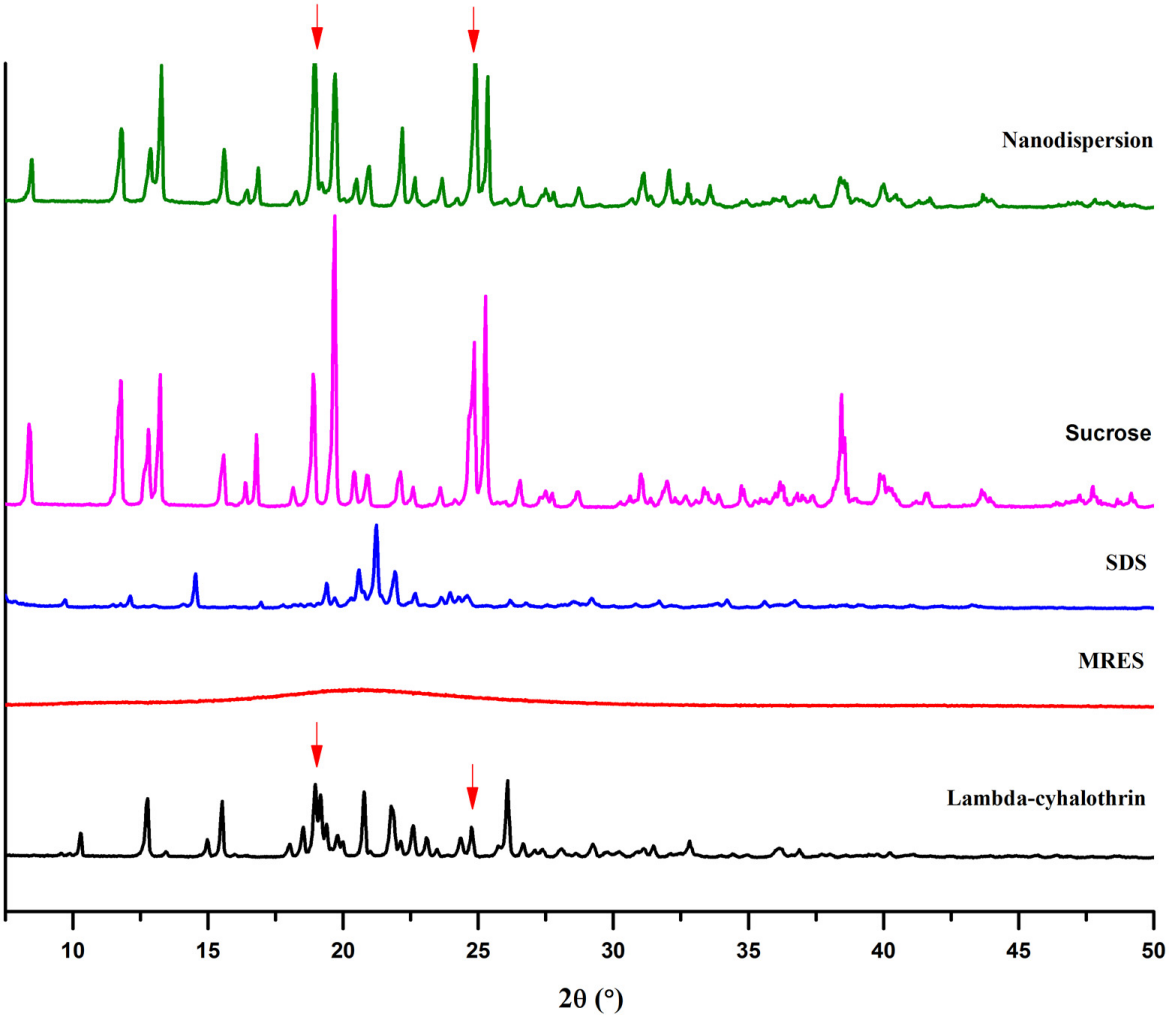
XRD patterns of pure components in the nano-formulation and lambda-cyhalothrin solid nanodispersion. SDS: 1-dodecanesulfonic acid sodium salt; MRES: maleic rosin-polyoxypropylene-polyoxyethylene ether sulfonate.

As shown in Fig 6, the XRD pattern of the solid nanodispersion presented characteristic peaks of crystalline lambda-cyhalothrin at 12.8°, 15.6°, 18.9°, 20.9° and 26.0°. In addition, diffraction peaks of the pesticide at 18.9° and 24.9° (labelled by red arrows) caused an increase of the corresponding peak intensity of the solid nanodispersion compared with the sucrose pattern. These results indicate that the crystallinity of the pesticide was preserved during the nanosizing process. In contrast, some wet-milling and high pressure homogenization processes may change material’s structure, even inducing progressive amorphization due to the large amount of heat and energy generated during the processes [23,39,40]. Furthermore, it was found that the first five intense peaks of the solid nanodispersion were mainly derived from crystalline sucrose. A reasonable explanation was the high content of sucrose in the mixture. According to the literature [41,42], the crystalline state improves stability during storage compared with the amorphous form, which increases molecular mobility and leads to aggregation issues.

#### 3.3.3. Suspensibility

The suspensibility of the solid nanodispersion in water was measured according to CIPAC MT 184 and calculated by the following equation:

$$\text{Suspensibility}(\%)=\frac{10}{9}\times \frac{{\text{m}}_{1}-{\text{m}}_{2}}{{\text{m}}_{1}}\times 100$$

Here, m_1_ and m_2_ are the pesticide contents of the original suspension and the remaining 25 ml of solution at the bottom, respectively. The suspensibilities of conventional pesticide formulations including WP, suspension concentrate (SC) and water dispersible granule (WDG) are usually less than 98%, even when additives are used to improve the performance [43–45]. It has been reported that suspensibility is inversely proportional to particle size [46]. Indeed, the suspensibility of the solid nanodispersion dispersed in water was up to 99.5%, as the particle size of lambda-cyhalothrin was less than 22 nm. The high suspensibility of the solid nanodispersion is favourable for improving the dispersibility and bioavailability of poorly water-soluble pesticides during field spraying after diluting with water.

#### 3.3.4. Wettability

Wettability is an important factor affecting pesticide adsorption, adhesion and efficacy. It relates to the ability of powder to be wetted or dispersed not only in liquid but also on leaves. The wettability of the lambda-cyhalothrin solid nanodispersion in water was evaluated according to CIPAC MT 53. The wetting time of pesticide WPs is generally longer than 50 s [47,48]. By contrast, the solid nanodispersion of lambda-cyhalothrin was completely wetted by water within 25 s, half that required by the WP formulation, which proves its good wettability.

The wettability of the solid nanodispersion was also evaluated by contact angle assessment on cucumber (*Cucumis sativus* L.) and rice (*Oryza sativa* L.) leaves. The contact angle of pure water on hydrophilic cucumber (*Cucumis sativus* L.) and hydrophobic rice (*Oryza sativa* L.) leaves was measured to be 84 ± 4° and 136 ± 3°, respectively, consistent with previous reports [49,50]. By contrast, the contact angles of the nanosuspension containing 0.5% (w/w) pesticide on these leaves were 58 ± 2° and 120 ± 4°, respectively, indicating better wettability. According to the literature [50], a possible explanation is that the surfactants as wetting agents in the composition of the solid nanodispersion played a crucial role in changing the surface wettability. Moreover, particle size reduction may have also contributed to the enhanced hydrophilicity by increasing the water solubility [51,52].

#### 3.3.5. Storage Stability

**Fig 7:**
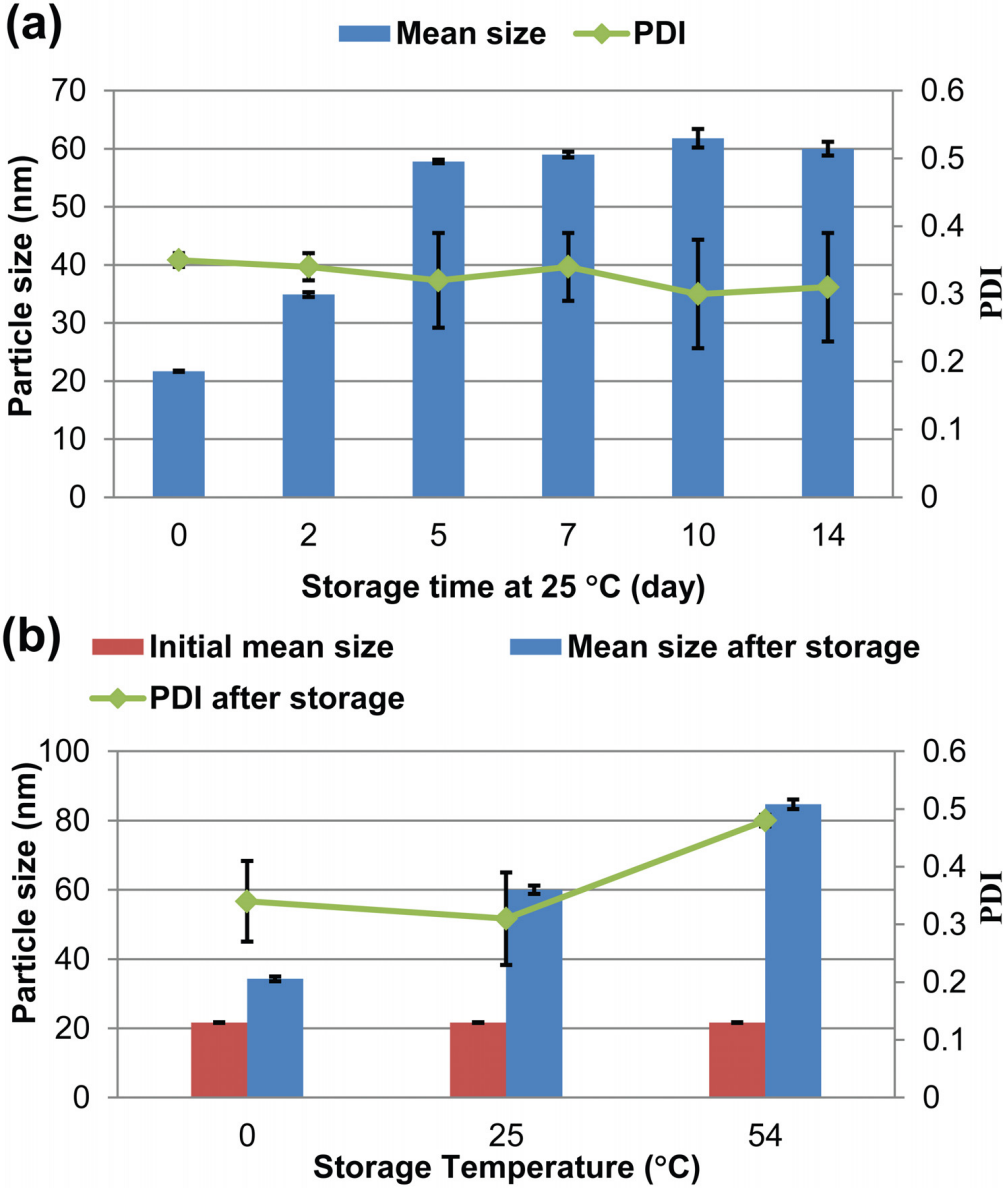
Stability of the lambda-cyhalothrin solid nanodispersion for (a) different storage times and (b) different storage temperatures. PDI: polydispersity index.

A stability test of the solid nanodispersion was conducted according to CIPAC MT 46 and GB/T 19136–2003. Fig 7a shows the change in particle size over a 14-day storage period at 25°C. Within five days, the particle size increased from 21.7 nm to 57.8 nm, and it then remained almost constant up to 14 days. In addition, the suspensibility and wettability of the nanodispersion after 14 days of storage were 99.4% and 23 s, respectively. This result illustrates that, after storage, the solid nanodispersion of lambda-cyhalothrin remained its good dispersibility and wettability relative to the WP formulation [53]. As shown in Fig 7b, the particle size of the nanodispersions increased to some extent but remained less than 85 nm after 14 days of storage at low (0°C), room (25°C) or high (54°C) temperatures. A similar particle growth phenomenon during storage has also been observed in other nano-formulations, such as microemulsions and nanosuspensions, due to Ostwald ripening [20,38,54]. The above results indicate that the nanodispersion has excellent storage stability. Moreover, the solid nanodispersion showed better stability under low and room-temperature storage than under high- temperature storage. One possible reason is the occurrence of nanoparticle aggregation and an increase in particle size at 54°C, which is above the melting point of lambda-cyhalothrin.

#### 3.3.6. Biological Activity

The results of a bioassay of the lambda-cyhalothrin solid nanodispersion are shown in Table 3. The LC 50 of the solid nanodispersion was lower than that of technical (TC), commercial EW and EC formulations. The toxicity of the solid nanodispersion to diamondback moths (*Plutella xylostella* L.) was 1.4, 1.2 and 1.1 times that of TC, EW and EC formulations, respectively. It has been reported that nano-pesticides have a greater and longer-lasting impact on target organisms than TC but their pesticidal activity was lower than 1.4 times of TC [55,56]. EC is widely regarded as an effective pesticide formulation, and its pest mortality could resemble that of nano-formulation in novaluron system [35,57]. The above results suggest that the solid nanodispersion of pesticide exhibited higher toxicity than conventional formulations and may be more promising than some other nano-formulations. The large specific surface area of pesticide nanoparticles could increase their retention on leaf surfaces and the contact area between target pests and the active ingredient [58,59], further improving the bioavailability of the pesticide nanodispersion.

**Table 3:** Bioassay results of four lambda-cyhalothrin formulations.

Formulation	Toxicity regression equation	Correlation coefficient	LC 50^a^ (μg/mL)	95% confidence limit
Solid nanodispersion	y = -2.1041 + 2.3253x	0.9671	1135.42	787.53~1637.01
TC^b^	y = 2.2879 + 0.8435x	0.9289	1641.63	828.48~3252.88
EW^c^	y = -2.7932 + 2.4957x	0.9593	1326.30	849.00~2071.93
EC^d^	y = -11.5156 + 5.3027x	0.9436	1301.91	769.35~2203.13

^a^LC 50: median lethal concentration.

^b^TC: technical.

^c^EW: emulsion in water.

^d^EC: emulsifiable concentrate.

## Conclusion

A novel formulation of lambda-cyhalothrin, a solid nanodispersion, was successfully prepared by a combination of melt-emulsification and high-speed shearing. The preparation method of this formulation has the benefits of simplified processing and reduced cost compared to the commonly used wet-milling and high pressure homogenization. The formulation characteristics of the solid nanodispersion with 21.7-nm-diameter particles, such as dispersibility, stability and bioavailability, were superior to those of the conventional pesticide formulations, thereby improving the pesticide’s efficacy. Moreover, the solid nanodispersion overcomes the disadvantages of conventional aqueous formulations, such as a short shelf life and inconvenient transportation. More importantly, the formulation is environmentally friendly due to its lack of organic solvents and the reduced use of surfactant in the composition. Therefore, the application of solid nanodispersions in crop production is helpful to reduce the residues in food as well as environmental pollution from pesticides.
